# Efficacy of calcium replacement in hypocalcaemia

**DOI:** 10.1186/cc12387

**Published:** 2013-03-19

**Authors:** H Chatha, K Sim

**Affiliations:** 1 Whiston Hospital, Liverpool, UK

## Introduction

The aim of the study is to examine the efficacy of calcium replacement in patients with hypocalcaemia in the ICU. Hypocalcaemia affects between 15 and 50% of admissions to the ICU [1].

## Methods

Adjusted calcium results <2.0 mmol/l were followed up from admission for 5 days or until discharge or death. On each day the adjusted calcium result and the calcium replacement were documented. Adequate replacement is defined as 40 ml of 10% calcium gluconate if the adjusted calcium is <2.0 mmol/l and 20 ml of 10% calcium gluconate if the adjusted calcium result is between 2.0 and 2.15 mmol/l.

## Results

Eighty-eight adjusted calcium results were <2.0 mmol/l. In 74 results adequate replacement was given and the average increase in calcium was 0.2 mmol/l. This led to an increase in the adjusted calcium to >2.15 mmol/l in 36% of results and improvement to >2.0 mmol/l in 89% of results. In 14 results no replacement was given and the average increase in calcium was 0.07 mmol/l, which equated to 57% of calcium results remaining <2.0 mmol/l. Unpaired *t *test showed a *P *value of 0.0006. A total of 150 adjusted calcium results were between 2.0 and 2.15 mmol/l. In 111 results adequate replacement was given and the average increase was 0.006 mmol/l. The adjusted calcium fell to <2.0 mmol/l in 26% of results and normalised to >2.15 mmol/l in 23%. Where the adjusted calcium fell despite replacement, 51% of results were below 2.05 mmol/l. In 39 results no replacement was given, leading to an average fall in calcium of 0.03 mmol/l. Thirty-three percent of calcium results fell to <2.0 mmol/l and 20% of results normalised. Unpaired *t *test showed a *P *value of 0.07.

## Conclusion

Calcium replacement with 40 ml of 10% calcium gluconate is effective when the adjusted calcium is <2.0 mmol/l. Replacement with 20 ml of 10% calcium gluconate when the adjusted calcium is between 2.0 and 2.15 mmol/l is not much more effective than no replacement. This suggests that either replacement is not needed or there is under-replacement in mild hypocalcaemia. Adjusted calcium may be giving falsely low calcium results in mild hypocalcaemia. The measurement of ionised calcium is a possible solution [2,3].
